# Protein dynamics at Eph receptor-ligand interfaces as revealed by crystallography, NMR and MD simulations

**DOI:** 10.1186/2046-1682-5-2

**Published:** 2012-01-25

**Authors:** Haina Qin, Liangzhong Lim, Jianxing Song

**Affiliations:** 1 Department of Biological Sciences, Faculty of Science, National University of Singapore, 10 Kent Ridge Crescent, Singapore 119260, Republic of Singapore; 2 Department of Biochemistry, Yong Loo Lin School of Medicine, National University of Singapore, 10 Kent Ridge Crescent, Singapore 119260, Republic of Singapore

## Abstract

**Background:**

The role of dynamics in protein functions including signal transduction is just starting to be deciphered. Eph receptors with 16 members divided into A- and B- subclasses are respectively activated by 9 A- and B-ephrin ligands. EphA4 is the only receptor capable of binding to all 9 ephrins and small molecules with overlapped interfaces.

**Results:**

We first determined the structures of the EphA4 ligand binding domain (LBD) in two crystals of P1 space group. Noticeably, 8 EphA4 molecules were found in one asymmetric unit and consequently from two crystals we obtained 16 structures, which show significant conformational variations over the functionally critical A-C, D-E, G-H and J-K loops. The 16 new structures, together with previous 9 ones, can be categorized into two groups: closed and open forms which resemble the uncomplexed and complexed structures of the EphA4 LBD respectively. To assess whether the conformational diversity over the loops primarily results from the intrinsic dynamics, we initiated 30-ns molecular dynamics (MD) simulations for both closed and open forms. The results indicate that the loops do have much higher intrinsic dynamics, which is further unravelled by NMR H/D exchange experiments. During simulations, the open form has the RMS deviations slightly larger than those of the closed one, suggesting the open form may be less stable in the absence of external contacts. Furthermore, no obvious exchange between two forms is observed within 30 ns, implying that they are dynamically separated.

**Conclusions:**

Our study provides the first experimental and computational result revealing that the intrinsic dynamics are most likely underlying the conformational diversity observed for the EphA4 LBD loops mediating the binding affinity and specificity. Interestingly, the open conformation of the EphA4 LBD is slightly unstable in the absence of it natural ligand ephrins, implying that the conformational transition from the closed to open has to be driven by the high-affinity interaction with ephrins because the weak interaction with small molecule was found to be insufficient to trigger the transition. Our results therefore highlight the key role of protein dynamics in Eph-ephrin signalling and would benefit future design of agonists/antagonists targeting Eph receptors.

## Background

The erythropoietin-producing hepatocellular carcinoma (Eph) receptors constitute the largest family of receptor tyrosine kinases, with 16 members throughout the animal kingdom, which are activated by 9 ephrin ligands [1-6]. Eph receptors and their ephrin ligands are both anchored onto the plasma membrane, which are subdivided into two subclasses, (A and B), based on their sequence conservation and binding preferences. In general, EphA receptors (EphA1-A10) only interact with glycosylphosphatidylinositol (GPI)-anchored ephrin-A ligands (ephrin-A1-A6), while EphB receptors (EphB1-B6) interact with transmembrane ephrin-B ligands (ephrin-B1-ephrin-B3) that have a short cytoplasmic domain carrying both SH2 and PDZ domain-binding motifs [7,8]. Interactions between Eph receptors and ephrins initiate bidirectional signals which direct pattern formation and morphogenetic processes, such as axon growth, cell assembly and migration, and angiogenesis [1-8]. The roles of Eph receptors and ephrins in bone remodelling, immune function, and blood clotting, and stem cells, are also starting to be characterized.

All Eph receptors share the same modular structure, consisting of a unique N-terminal ephrin binding domain followed by a cysteine-rich linker and two fibronectin type III repeats in the extracellular region. The intracellular region is composed of a conserved tyrosine kinase domain, a C-terminal sterile α-domain, and a PDZ binding motif. The N-terminal 180-residue globular domain of the Eph receptors has been shown to be sufficient for high-affinity ephrin binding [9-11], thus called the ligand binding domain (LBD). So far, structures have been determined for the Eph LBD in the free state [9,12-15], in the complexed forms between A-receptors and A-ephrins [12,13,16,17]; A-receptors and B-ephrins [13,18]; B-receptors and B-ephrins [11,19] and B-receptors and A-ephrins [20], as well as between receptors and antagonistic peptides [21,22]. The ligand binding domains of both EphA and EphB receptors adopt the same jellyroll β-sandwich architecture composed of 11 antiparallel β-strands connected by loops of various lengths. On the other hand, the ectodomain of the ephrins is also conserved and consists of an eight-stranded β-barrel with a Greek key topology, including several large and highly conserved functional loops, such as the G-H and C-D loops [11-18], which are highly dynamic in solution as revealed by a NMR study [23]. The common structural feature observed in Eph-ephrin complexes is the insertion of the solvent-exposed and dynamic ephrin G-H loop into the Eph receptor hydrophobic channel formed by the convex sheet of four β-strands capped by the D-E, J-K, and G-H loops. Nevertheless, additional interactions such as the involvement of the A-C loop fine-tune the affinity and specificity of the binding cross subclasses [18].

Interactions between the Eph receptors and ephrins of the same subclass are quite promiscuous but interactions between subclasses are relatively rare. EphA4 is the only receptor capable of interacting with all 9 ephrins of both A- and B-subclasses to mediate a diverse spectrum of biological activities [24]. While EphA4 interacts with ephrin-A ligands to mediate a variety of critical biological processes, such as inhibiting integrin downstream signaling pathways and modulating sensory and motor projections [25-27], it is also able to bind all three ephrin-B ligands. For example, EphA4 interacts with ephrin-B1 expressed in human platelets to stabilize blood clot formation through an integrin-dependent mechanism [28]. By interacting with ephrin-B2 and/or ephrin-B3, EphA4 regulates neuronal circuits important for coordinated movement and may inhibit the regeneration of injured spinal cord axons [29-31]. As a consequence, EphA4 was also considered as a promising target for the development of small molecule drugs to treat human diseases [14,32].

The unique ability for the EphA4 LBD to bind all 9 ephrins with similar interfaces renders it to be an attractive model for deciphering the fundamental principle governing protein-ligand interactions. Currently, our understanding of molecular recognition is still incomplete, and in particular the role of protein dynamics in mediating binding affinity and specificity remains to be delineated. Previously, 9 crystal structures were determined for the EphA4 LBD in the free state [13-15] and in complex with ephrinA2 and ephrinB2 [13,18]. The most outstanding observation is that while the jellyroll β-sandwich core is highly similar in all these structures, the loops, especially D-E, G-H and J-K loops critical for binding, have dramatic conformational variations, which is largely unexpected for such a small protein [15]. This implies that the functionally critical loops might have higher dynamics but it remains to be clarified that the variations of loop conformations are not primarily due to the crystal packing force or/and differential crystallization conditions.

In the present study, we obtained two crystals of the EphA4 LBD at P_1 _space group and subsequently determined their structures. Remarkably, 8 EphA4 chains were found in one asymmetric unit and as a result we gained 16 new structures from the two crystals. Although the 16 structures have an almost identical conformation over the jellyroll β-sandwich core, they display significant variations over the A-C, D-E, G-H and J-K loops, which consequently led to the classification of the 16 structures into two groups: closed and open forms based on the conformations of the D-E and J-K loops. To gain insight into the dynamical behaviours and the relationship between the closed and open forms, we initiated 30-ns molecular dynamics (MD) simulations for two structures, which represents the closed and open forms respectively. The obtained results show that indeed the loops do have much larger intrinsic dynamics than the rest of the molecules, which was further supported by NMR hydrogen-deuterium (H/D) exchange experiments.

## Results

### Structure determination

Previously, the crystal structures of the uncomplexed EphA4 LBD have been determined in space groups of P2_1 _[13], P22_1_2_1 _[14] and P2_1_2_1_2_1 _[15]. In the present study, we obtained two crystals of the EphA4 LBD with the space group of P_1 _and subsequently determined their structures at 2.6 and 3.0 Å respectively, by molecular replacement with the search model generated from our previous structure of the free EphA4 LBD (3CKH). Table 1 summarizes the details of the data collection and refinement statistics.

**Table 1:** Crystallographic data and refinement statistics for the EphA4 structures

Data collection	Crytal1	Crystal2
Wavelength (Å)	1.5418	1.5418
Resolution limit (Å)	50-2.9	50-2.4
Space group	P1	P1
**Cell parameters**		
a, b, c (Å)	53.212, 70.621, 126.985	46.881, 70.030, 123.103
α, β, γ (°)	90.011, 90.036, 89.999	89.982, 89.972, 89.990
Unique reflections	75555	98655
Completeness	97.7%	94.6
Redundancy	1.9	1.8
Linear R-factor	0.473	0.094
**Refinement**		
Resolution range (Å)	25.0-3.0	25.0-2.6
R _work_	0.236	0.237
Number of Reflections/test	34290/1816	48082/4071
R_free_	0.312	0.262
Rmsd bond lengths (Å)	0.013	0.011
Rmsd bond angles (deg)	1.613	2.063
**Ramachandran plot**		
Favored, %	75.9	72.9
Allowed, %	18.2	21.4
Generously allowed, %	3.5	3.2
Disallowed, %	2.4	2.4

**Figure 1:**
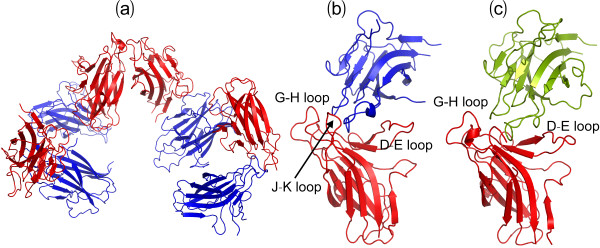
**Crystal structures of the EphA4 LBD**. (a) Packing relationship of 8 structures of the EphA4 LBD in one asymmetric unit of P_1 _space group. The EphA4 molecule with (red) or without (blue) its high affinity ephrin binding pocket closely contacting the G-H loop of another EphA4 molecule in the same (b) or a neighboring (c) unit.

Most distinguishably, 8 EphA4 chains were found in one asymmetric unit (Figure 1a) and consequently we obtained 16 new crystal structures from two crystals. A close examination reveals that in the asymmetric unit, 8 molecules have differential packing. As exemplified by the asymmetric unit of the crystal 1 (Figure 1a), while the high-affinity ephrin-binding pocket of 4 EphA4 molecules in blue shows no close contact with other molecules, those of other 4 in red have tight contacts with the G-H loop of another molecule either in the same (Figure 1b) or neighbouring units (Figure 1c). In the asymmetric unit of the crystal 2, 8 EphA4 molecules display a slightly-different packing relationship from the crystal 1 and consequently only one molecule has its high-affinity ephrin-binding pocket closely contacting the G-H loop of another molecule (result not shown).

### Loop conformations

**Figure 2:**
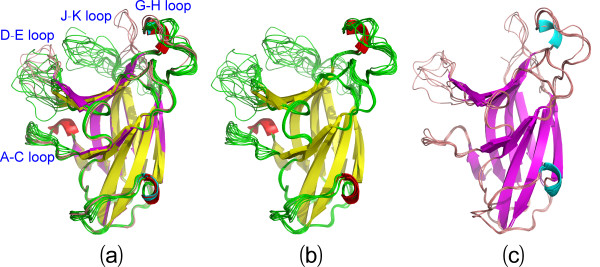
**Comparison of 16 structures**. (a) Superimposition of 16 structures of the EphA4 LBD, which can be divided into the closed and open forms based on the conformations of D-E and J-K loops. The color codes for Helix-Sheet-Loop are Red-Yellow-Green for the closed and Cyan-Purple-Brown for the open forms respectively. (b) Superimposition of 11 structures of the EphA4 LBD in the closed form. (c) Superimposition of 5 structures of the EphA4 LBD in the open form.

**Figure 3:**
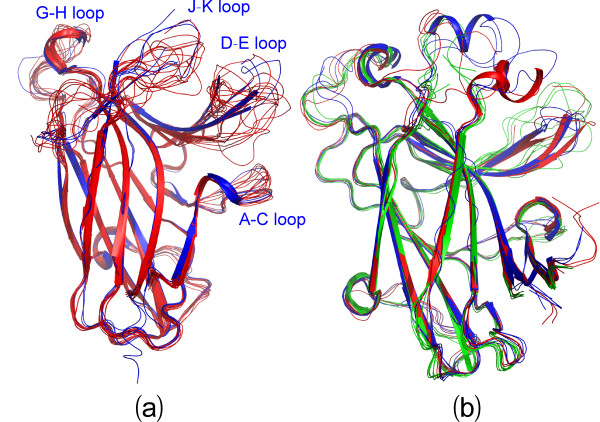
**Comparison with previously-determined structure**. (a) Superimposition of 11 present structures (red) with previously-determined 3 structures (blue; 3KH and 2WO1) of the EphA4 LBD in the closed form. (b) Superimposition of 5 present structures (green) with previously-determined 6 structures (3KH, 3GXU, 2WO2, 2WH3 and ref. [15]) of the EphA4 LBD in the open form. The open structures in the uncomplexed state are colored in red while the open structures complexed with ephrin are in blue.

Superimposition of 16 structures reveals a remarkable feature: while the jellyroll β-sandwich core of the EphA4 LBD has almost identical conformations, large conformational variations can be observed over loops, in particular over A-C, D-E, G-H and J-K loops (Figure 2a), all of which have been previously demonstrated to modulate the binding affinity and specificity to ephrins. Based on the conformations of the D-E and J-K loops, the 16 structures can be approximately categorized into two groups: the closed form containing 11 structures whose high-affinity ephrin-binding pocket has no close contact to other molecules (Figure 2b) and open form constituted by 5 structures whose high-affinity ephrin-binding pocket has close contacts to other molecules (Figure 2c). Strikingly, as seen in Figure 3a, the structures of the closed form are similar to those of the uncomplexed EphA4 LBD whose high-affinity ephrin-binding pocket has no close contact to other molecules [13,14]. On the other hand, as shown in Figure 3b, the structures of the open form resemble those of the EphA4 LBD either in complex with ephrins [13,18], or in the free state whose high-affinity ephrin-binding pocket has close contacts to other molecules [14,15]. It is also worthy of pointing out that even within the same form, different structures have the loop conformations variable to some degree, particularly for the A-C, D-E, G-H and J-K loops.

### Molecular dynamics (MD) simulations

**Figure 4:**
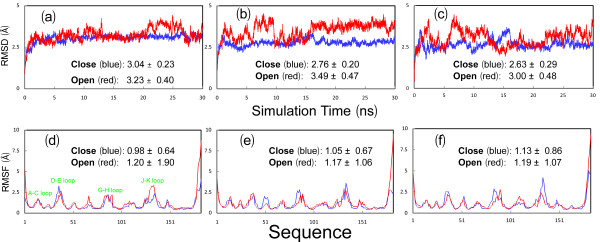
**Trajectories of MD simulations**. (a-c). Root-mean-square deviations (RMSD) of the heavy atoms for three independent MD simulations for the closed (blue) and open (red) forms. (d-f). Root-mean-square fluctuations (RMSF) of the Cα atoms computed for three independent simulations for the closed (blue) and open (red) forms. The average values and standard deviations over 30-ns simulations are computed and displayed.

To explore their dynamical behaviours, we initiated 30-ns MD simulations for both closed and open structures. Figures 4a-c present the root-mean-square deviations (RMSD) of the heavy atoms for three parallel simulations. It appears that for all simulations, the RMSD values increased very rapidly during the first 0.8 ns. This is mostly due to the relaxations of the crystal structures upon being solvated in solution. Very strikingly, the two forms display a slight difference in overall dynamic stability. The closed form has RMSD values of 3.04 ± 0.23, 2.76 ± 0.20 and 2.63 ± 0.29 Å respectively for three independent simulations. By contrast, the open form shows the slightly higher overall conformational flexibility and fluctuation, with the RMSD values of 3.23 ± 0.40, 3.49 ± 0.47 and 3.00 ± 0.48 Å respectively. This is likely due to the possibility that the open form would become overall unstable upon losing the contacts with the G-H loop of another EphA4 molecule as observed in the crystal (Figure 1).

### Detailed dynamical behaviours

Figures 4d-f present the root-mean-square fluctuations (RMSF) of the Cα atoms in three MD simulations for both closed and open forms of the EphA4 LBD. Consistent with the overall dynamical behaviours captured by the RMSD trajectories (Figures 4a-c), the closed form has the slightly less overall conformational fluctuation, with RMSF values of 0.98 ± 0.64, 1.05 ± 0.67 and 1.13 ± 0.86 Å respectively for three independent simulations, while the open form has the higher overall conformational fluctuation, with RMSF values of 1.20 ± 1.90, 1.17 ± 1.06 and 1.19 ± 1.07 Å respectively.

**Figure 5:**
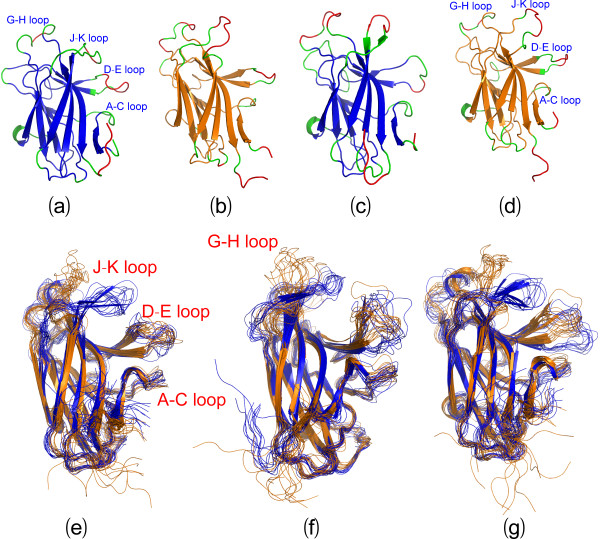
**Detailed dynamical behaviours**. The closed (a and c) and open (b and d) structures of the EphA4 LBD in which green is used for coloring residues with their RMSF values > average value and red for the residues with their RMSF values > 2 average value. (e-g) Structure snapshots (one structure for 3-ns interval) of three independent MD simulations respectively for the closed (blue) and open (brown) forms.

On the other hand, examination of the fluctuations of the Cα atoms over the sequence indicates that the closed and open forms have similar overall patterns. More precisely, for the closed form in the first simulation, the residues with RMSF values > its average value are located on two termini and loops, including residues Gly1-Asn3 on N-terminus, Arg11-Gly18 on A-C loop, Ser32-Arg42 on D-E loop, Pro52-Ser53 on E-F loop, Gly67 on F-G loop, Asn83-Thr91 on G-H loop, Arg109-Asn114 on H-I loop, Asp125-Met138 on J-K loop, Gly148-Pro149 on K-L loop and Lys176-Arg183 on C-terminus. Furthermore, the residues with RMSF values > 2 average value only include Met34-Thr39 on the tip of D-E loop, Pro86-Gly87 on G-H loop, Gly134-Asp135 on J-K loop and Pro179-Arg183 on C-terminus. Although slight differences are observed in other two simulations, their overall patterns of the RMSF trajectories are quite similar to that of the first simulation for the closed form (Figures 4d-f, 5a-d).

**Figure 6:**
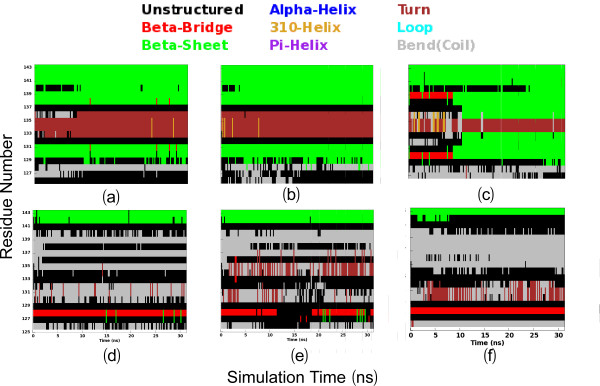
**Trajectories of secondary structures of the J-K loop residues**. Three independent trajectories of secondary structures of the J-K loop residues (125-143) of the closed (a-c) and open (d-f) forms during 30-ns simulations.

Figures 5e-g present the structure snapshots of both closed and open forms in three simulations respectively, which show the dramatic conformational fluctuations of loops in both forms. Strikingly, within the 30-ns simulations, the conformational ensembles of the D-E loop become similar in the closed and open forms, while those of the J-K loop are still considerably distinctive in two forms. It is particularly interesting to note that in the closed form there is a short β-sheet composed of the J-K residues Thr129-Gln130-Val131 and Ile137-Met138-Lys139 (Figures 5e-g), which persists in almost all 30-ns trajectories; and in some time intervals this sheet even becomes longer (Figures 6a-c). By contrast, in the open form the corresponding residues have no regular secondary structure in 30-ns trajectories (Figures 6d-f). This observation is completely consistent with our NMR result that for the free EphA4 LBD in solution, these residues indeed form a short β-sheet [14]. The above results together strongly imply that the closed and open conformations are not only structurally distinguishable, but also dynamically separated.

**Figure 7:**
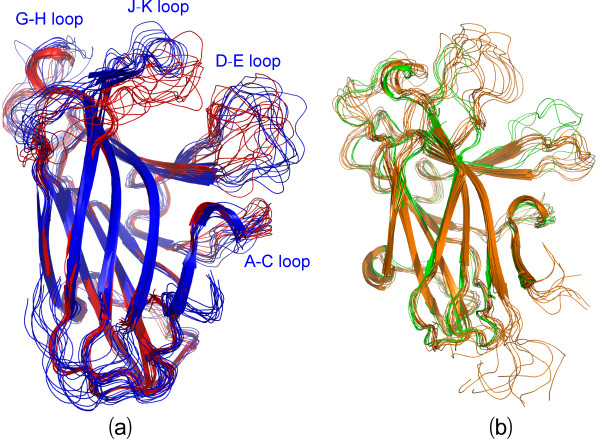
**Comparison between crystal structures and simulation ensembles**. (a) Superimposition of 14 crystal structures (red) with 10 structure snapshots (blue, one structure for 3-ns interval) in the closed form. (b) Superimposition of 11 crystal structures (green) with 10 structure snapshots (brown, one structure for 3-ns interval) in the open form.

Interestingly, the loop conformations of the available crystal structures within the closed form appear to be within the structural ensemble of the 30-ns simulations for the representative closed structure (Figure 7a), suggesting that different closed conformations are exchangeable within 30 ns. Conversely, the crystal structures of the open form show large differences from the structural ensemble of the 30-ns simulations for the representative open structure (Figure 7b), implying that the formation of different open conformations might largely rely on the specific interactions with other molecules/ligands.

### H/D exchange experiments

**Figure 8:**
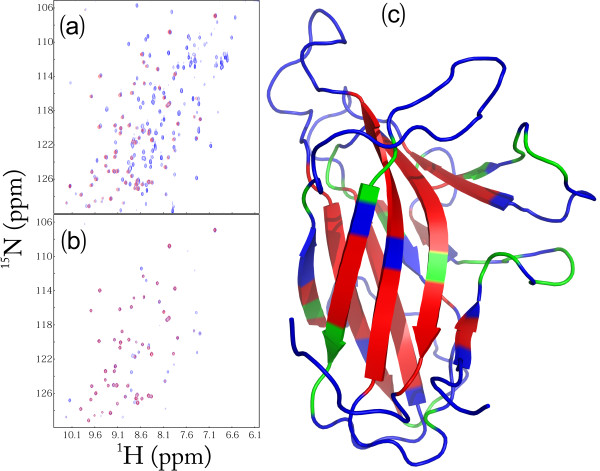
**NMR hydrogen-deuterium (H/D) exchange**. (a) Superimposition of the ^1^H-^15^N NMR HSQC spectra of the ^15^N-labeled EphA4 LBD at 25°C in the buffer (blue) and 15 min (red) after the lyophilized EphA4 LBD powder was re-dissolved in D_2_O. (b) Superimposition of the ^1^H-^15^N NMR HSQC spectra of the ^15^N-labeled EphA4 LBD at 25°C, 15 min (blue) and 2 hr (red) after the lyophilized EphA4 LBD powder was re-dissolved in D_2_O. (c) The structure of the EphA4 LBD with the H/D exchange results mapped onto. Blue: the residues completely exchanged within 15 min; green: residues completely exchanged from 15 min to 2 hr; red: residues un-exchanged after 2 hr.

We also utilized NMR hydrogen/deuterium (H/D) exchange and to assess the backbone dynamics of the EphA4 LBD on min-hr time scale. As well-established, in solution labile hydrogens such as amide protons on proteins are continually exchanging with the solvent at different rates, depending on a variety of factors associated with their environment including their exposure to the solvent or their involvement in H-bonds. Consequently, amide H/D exchange experiments offer a sensitive reflection of the exposure degree of amide protons to the solvent [33]. As seen in Figure 8a, upon subjecting to H/D exchange, ~59% of the total residues have completely exchanged with deuterium within the experimental dead time (15 min). These fast-exchange rate residues cover not only most residues on the loop and helical regions, but also some on the β-strands (Figure 8c). After 2.0 h, amide protons of more residues exchanged and consequently only ~27% of the total residues have persisted HSQC peaks (Figure 8b), which are mostly distributed on the β-strands (Figure 8c) and thus characterized as slow-exchange-rate residues. After 24 hr, several more HSQC peaks further disappeared (spectrum not shown). These results strongly indicate that the EphA4 LBD is also highly dynamic on the min-hr time scale, similar to what we have previously observed on the human ephrin-B2 [23].

## Discussion

Protein-ligand interactions play key roles in a variety of biological processes including enzymatic catalysis and signal transduction. As such, delineation of the principle mediating binding affinity and specificity is not only of fundamental interest, but also the prerequisite for rational design of molecules for therapeutic applications. Recently, accumulating evidence reveals that protein functions cannot be completely rationalized by the average three-dimensional structures determined by X-ray crystallography and NMR spectroscopy, and the key role of protein dynamics is just starting to be appreciated [34-40]. This can be nicely reflected by the evolution of the concept of binding mechanisms from the early 'lock-and-key' hypothesis [41], to the recently popular 'induced fit' [42-45] and 'conformational selection' models [34,37,38,46-49], in both of which protein dynamics play a central role.

Protein dynamics may be the underlying mechanism for the rare capacity of the EphA4 LBD to bind not only all 9 natural ligand ephrins/VAP-MSP, but also designed peptides [50], designed and endogenous small molecules [14,32,51]. The present determination of 16 new structures, together with previous 9 structures, reveal a surprising feature: the functionally critical A-C, D-E, G-H and J-K loops have very distinctive conformations in different crystal structures of the EphA4 LBD. This observation strongly implies that these loops might be more dynamic than the rest of the protein. In the present study, by MD simulations, the A-C, D-E, G-H and J-K loops of the EphA4 LBD have been demonstrated to have much higher intrinsic dynamics than the rest of the molecule on ns time scale. The H/D exchange results further denote that these loops are also highly dynamic and exposed to bulk solvent in the min-hr time scale. Therefore, our results reveal that the conformational diversity observed over these loops in the 25 crystal structures is primarily resulting from their intrinsic dynamics.

The existence of diverse conformations over the A-C, D-E, G-H and J-K loops even in the different crystal structures of the closed form strongly implies that the energy barriers separating them are small and these conformations might pre-exist in equilibrium for the EphA4 LBD in the free state. This is supported by the results that during the 30-ns simulations of the closed structure, the loops are able to sample an ensemble of conformations highly similar to those observed in different crystal structures. Our results thus support the recently-proposed scenario that the protein segments with key roles in mediating binding processes have dynamics much higher than the rest of the protein molecule, thus allowing rapid redistributions of pre-encoded conformational states which is central to the cellular signalling [37,48]. As for the EphA4 LBD, the pre-existence of these conformations may significantly facilitate the rapid response to small molecule ligands following the 'conformational selection' scenario [48] as their bindings require no dramatic conformational rearrangement [14].

On the other hand, the MD results also indicate that the open form is well separated from the closed one, which is evidenced from the persistence of the short β-sheet over the J-K loop characteristic of the closed form over the whole 30-ns simulations. This implies that the closed and open conformations are separated by relatively large barriers and consequently the transition from the closed to open form needs longer time, e.g. μs-ms. In other words, this transition has to be driven by the binding to ephrins with much higher affinity. As the transition characteristic of the disruption of the short β-sheet has been observed in all Eph-ephrin complex structures, here we speculate that while the ps-ns dynamics over the loops may be common to all Eph receptors, the EphA4 LBD is expected to have extensive conformational exchanges over μs-ms timescale which thus allow the selection of different pre-existing open conformations by different ephrins. However, the "induced fit" may also be involved in Eph-ligand interactions. Indeed, the distinction between the "induced fit" and "conformational selection" models appears not that absolute [37,52,53], and an increasing number of reports indicate that conformational selection is usually followed by conformational adjustment [54,55]. To test this hypothesis, we have initiated a systematic mapping of protein dynamics of EphA4 and several other Eph LBDs over both ps-ns and μs-ms time scales by NMR spectroscopy. In conjunction with MD simulations, we expect to have a better understanding how protein dynamics mediate the affinity and specificity for Eph-ligand interactions in the near future. With the availability of such knowledge, we may able to ultimately design agonists/antagonists targeting different conformational states of Eph receptors [48,49].

## Conclusions

In summary, in the present study, we determined 16 new crystal structures of the EphA4 LBD, which can be categorized into the closed and open forms. The 16 new structures together with previous 9 ones reveal an extreme conformational diversity over the functionally-important A-C, D-E, G-H and J-K loops. Furthermore, by using MD simulation and NMR H/D exchange experiments, we provided the strong evidence that the conformational diversity over the EphA4 loops is most likely resulting from their intrinsic dynamics. We have also proposed a dynamic scenario to rationalize the unique ability of the EphA4 LBD in binding all 9 ephrins, as well as peptides and small molecules.

## Methods

### Cloning and expression of the EphA4 LBD

The DNA fragment encoding the human EphA4 LBD over residues 28 to 208 was amplified from cDNA library of Hela cell line as we previously described [14]. The obtained gene was subsequently cloned into a modified pET32a vector (Novagen) with the S-tag and thioredoxin genes removed. The recombinant protein was over-expressed in Rosetta gama-B strain (Novagen) and induced by 0.1 mM isopropyl 1-thio-β-D-galactopyranoside (IPTG) overnight at 20°C. After cell harvest and lysis, the recombinant protein was purified by Ni^2+^-affinity column (Qiagen), followed by in-column cleavage by thrombin to separate the LBD from His-tag. The release protein was further purified by FPLC with column superdex G-75 (GE Healthcare). MALDI-TOF mass spectrometry was applied to verify the protein.

### Crystallization, Data Collection and Structure Determination

The EphA4 LBD was prepared at a concentration of 10 mg/ml and crystallized by setting up 2 μL hanging drops at room temperature in a well containing the reservoir solution (17% PEG 4000, 11% isopropanol, and 0.1 M HEPES, pH 7.5). Rock-like crystals formed after 7 days. X-ray diffraction images for a single crystal were collected by using an in-house Bruker X8 Proteum X-ray generator with a CCD detector. Two crystals were diffracted and the data were indexed and scaled by HKL2000 package [56]. After an all-space-group search, the crystals were identified to belong to the space group P_1 _with 8 EphA4 molecules per asymmetric unit. The Matthews coefficient was calculated as 2.98 with 58.78% solvent constant and 2.53 with 51.32% solvent constant respectively by CCP4 software package [57].

The structure was determined through the molecular replacement with the search model generated by using our previously determined structure of the free EphA4 LBD (3CKH) [14]. The refinement was carried out by program Refmac [56]. The details of the data collection and refinement statistics are shown in Table 1. All the figures were prepared using the PyMOL molecular graphics system (W. L. DeLano, DeLano Scientific LLC, San Carlos, CA).

### Molecular dynamics (MD) simulations

To unravel the intrinsic dynamic behaviors of the closed and open forms of the EphA4 LBD, three independent, 30-ns MD simulations were set up for each of them as we previously described on the SARS 3C-like protease and MSP [39,40]. Briefly, the simulation cell is a periodic cubic box with a minimum distance of 9 Å between the protein and the box walls to ensure the proteins would not directly interact with its own periodic image. The water molecules, described using the TIP3P model, were filled in the periodic cubic box for the all atom simulation. Each set of MD simulations was implemented by using the program GROMACS [58], with the AMBER 99SB-ILDN all-atom force field [59]. The long-range electrostatic interactions were treated using the fast particle-mesh Ewald summation method [60]. The temperature during simulations was kept constant at 300 K by Berendsen's coupling. The pressure was held at 1 bar. The isothermal compressibility was 4.6*10^-5 ^bar^-1^. The time step was set as 2 fs. All bond lengths including hydrogen atoms were constrained by the LINCS algorithm [61]. Prior to MD simulations, all the initial structures were relaxed by 5000 steps of energy minimization using steepest descent algorithm, followed by position restraint equilibration for 200 ps.

Time evolution of secondary structures of the EphA4 J-K loop residues (125-143) was analyzed by DSSP (Definition of Secondary Structure Prediction) program [62] for 30-ns MD simulations.

### NMR H/D exchange experiments

The hydrogen-deuterium (H/D) exchange experiments were conducted as we previously described on the human ephrinB2 [23] and MSP domain [40]. Briefly, the ^15^N-labeled EphA4 LBD in the 10 mM (pH 6.3) phosphate buffer was lyophilized and then re-dissolved in D_2_O. Progress of the exchange process between amide protons and deuterium was followed by collecting a series of successive HSQC spectra starting immediately after the sample re-solubilization in D_2_O. All exchange experiments were conducted on an 800 MHz Bruker Avance spectrometer at 25°C. The first HSQC spectrum was collected after 15 min, and the last spectra were acquired after 24 h.

## Authors' contributions

QHN participated in design and carried out experiments and analysis for crystallography and NMR studies; and writing up the manuscript. LLZ performed MD simulations and analysis. SJX conceived of the study, participated in design, result analysis, coordination and writing up the manuscript. All authors read and approved the final manuscript.
